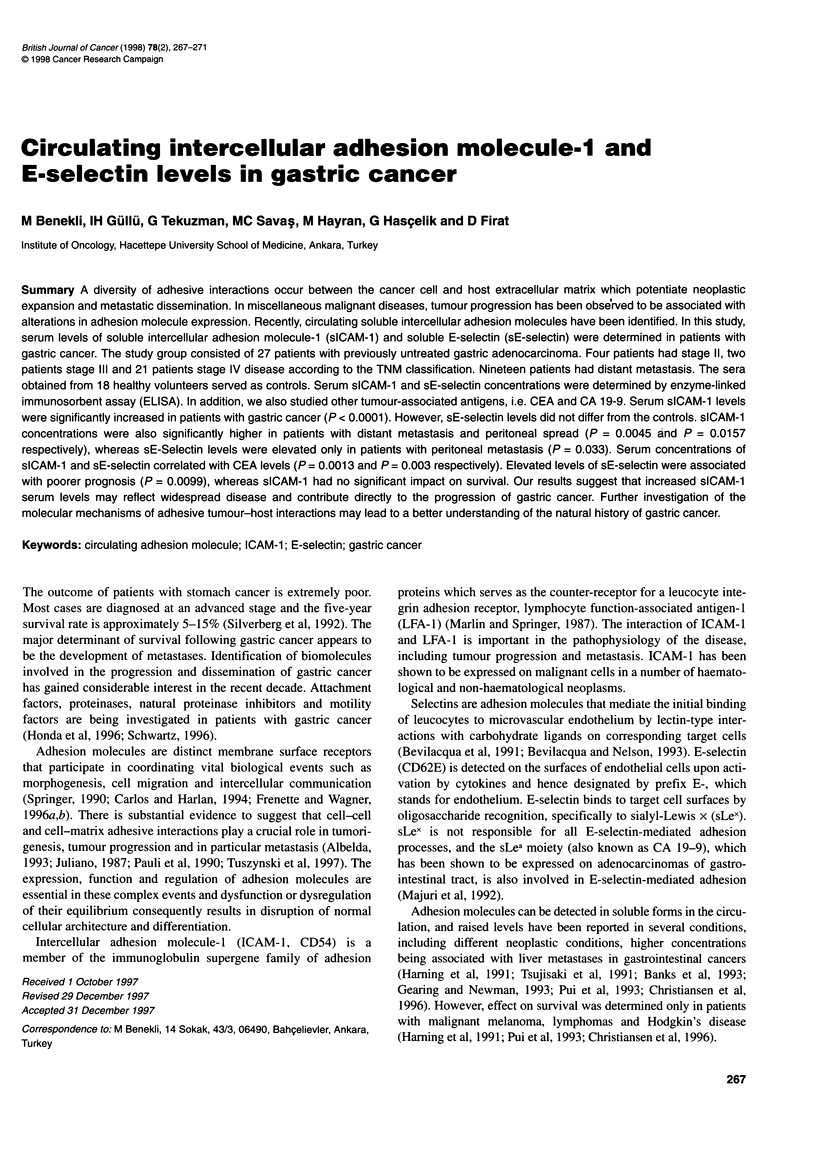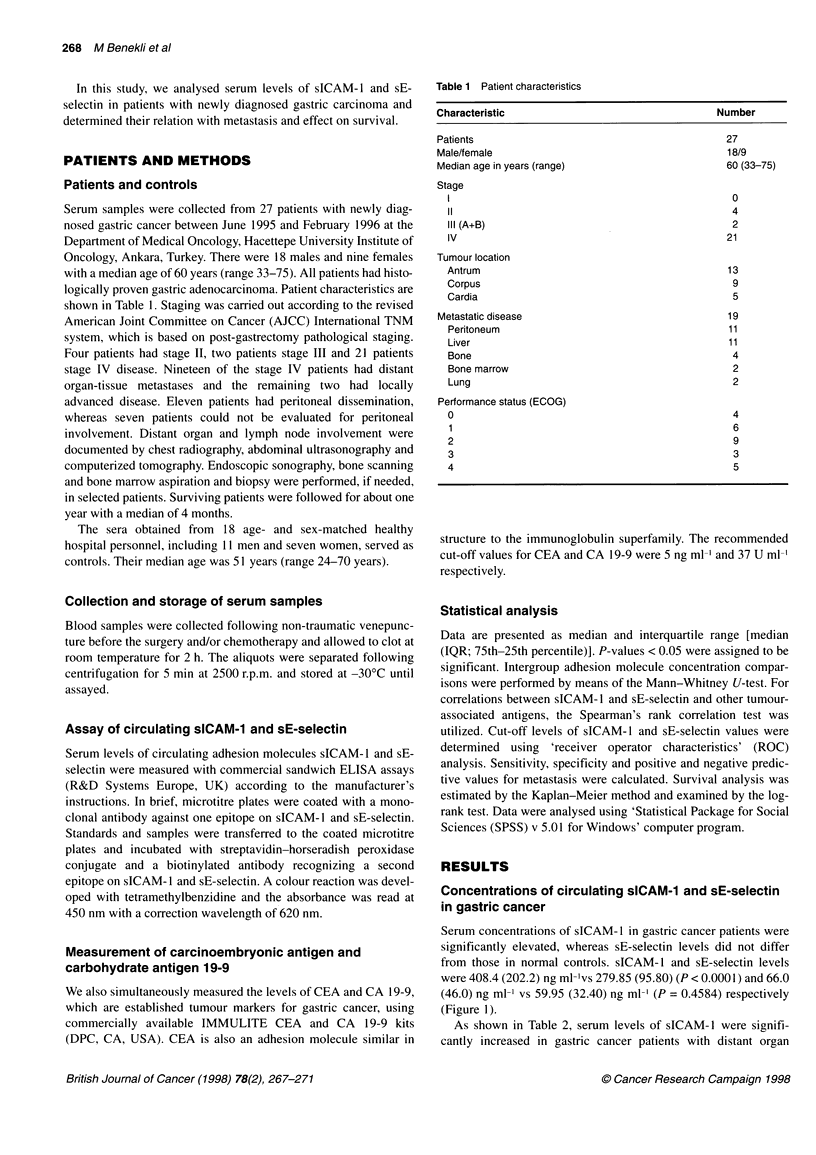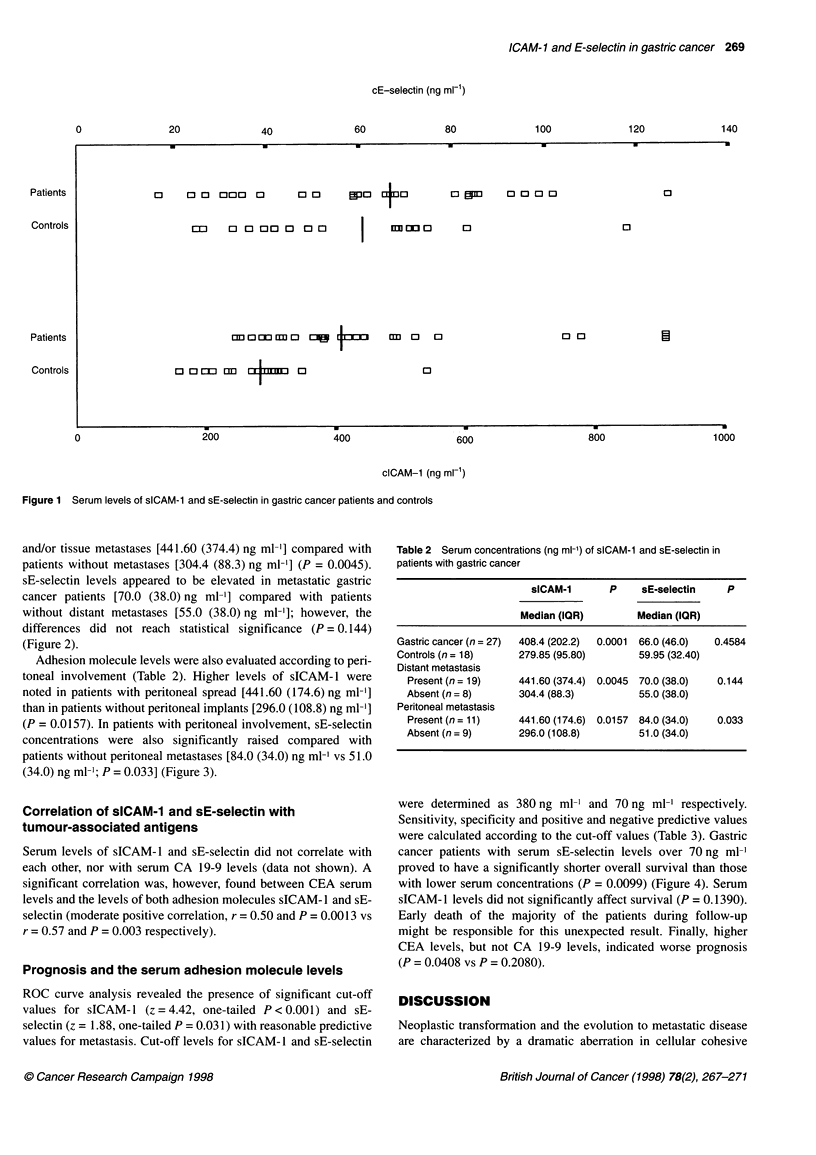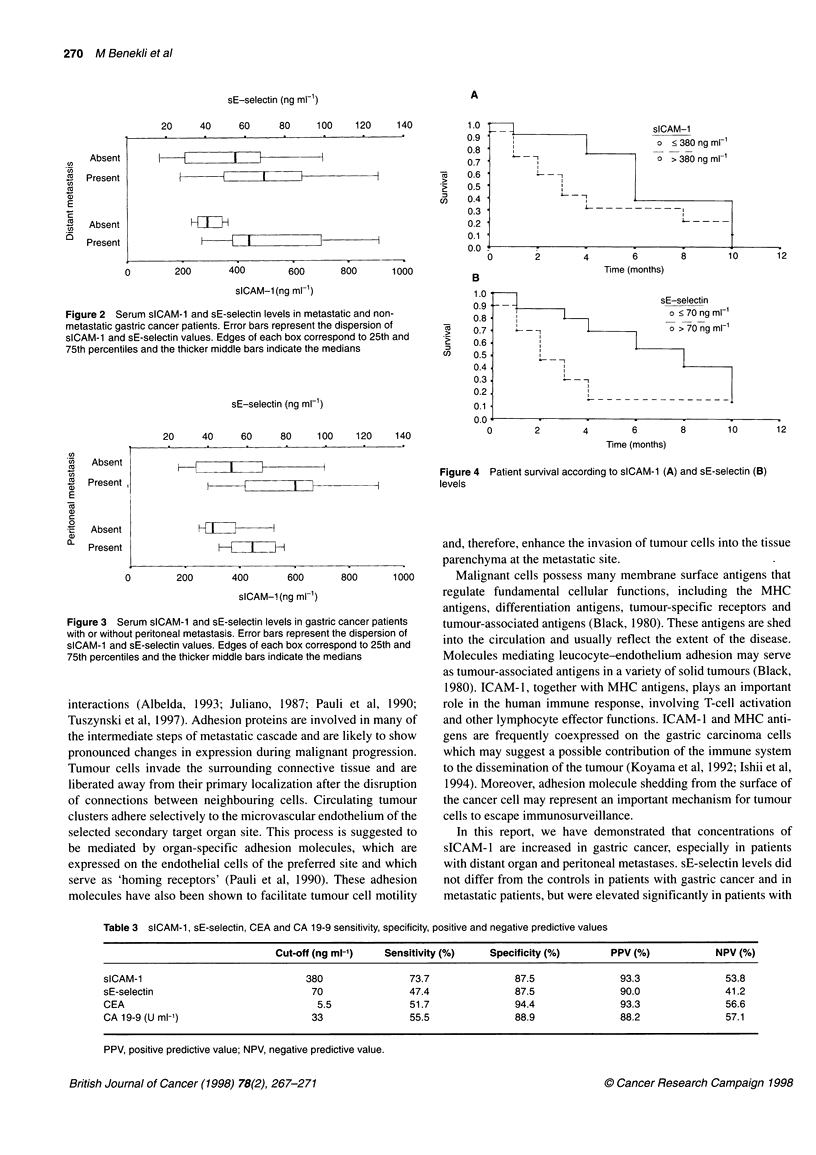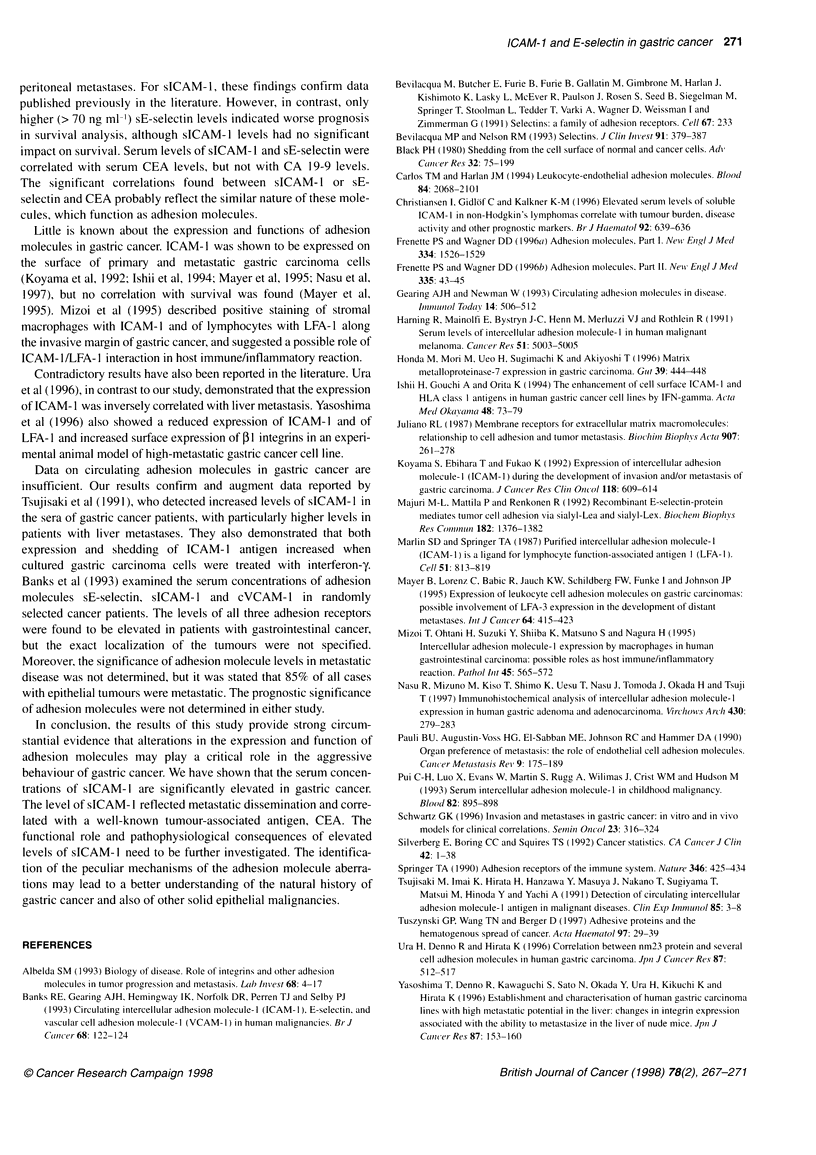# Circulating intercellular adhesion molecule-1 and E-selectin levels in gastric cancer.

**DOI:** 10.1038/bjc.1998.476

**Published:** 1998-07

**Authors:** M. Benekli, I. H. Güllü, G. Tekuzman, M. C. Savaş, M. Hayran, G. Hasçelik, D. Firat

**Affiliations:** Institute of Oncology, Hacettepe University School of Medicine, Ankara, Turkey.

## Abstract

A diversity of adhesive interactions occur between the cancer cell and host extracellular matrix which potentiate neoplastic expansion and metastatic dissemination. In miscellaneous malignant diseases, tumour progression has been observed to be associated with alterations in adhesion molecule expression. Recently, circulating soluble intercellular adhesion molecules have been identified. In this study, serum levels of soluble intercellular adhesion molecule-1 (sICAM-1) and soluble E-selectin (sE-selectin) were determined in patients with gastric cancer. The study group consisted of 27 patients with previously untreated gastric adenocarcinoma. Four patients had stage II, two patients stage III and 21 patients stage IV disease according to the TNM classification. Nineteen patients had distant metastasis. The sera obtained from 18 healthy volunteers served as controls. Serum sICAM-1 and sE-selectin concentrations were determined by enzyme-linked immunosorbent assay (ELISA). In addition, we also studied other tumour-associated antigens, i.e. CEA and CA 19-9. Serum sICAM-1 levels were significantly increased in patients with gastric cancer (P < 0.0001). However, sE-selectin levels did not differ from the controls. sICAM-1 concentrations were also significantly higher in patients with distant metastasis and peritoneal spread (P = 0.0045 and P = 0.0157 respectively), whereas sE-Selectin levels were elevated only in patients with peritoneal metastasis (P = 0.033). Serum concentrations of sICAM-1 and sE-selectin correlated with CEA levels (P = 0.0013 and P = 0.003 respectively). Elevated levels of sE-selectin were associated with poorer prognosis (P = 0.0099), whereas sICAM-1 had no significant impact on survival. Our results suggest that increased sICAM-1 serum levels may reflect widespread disease and contribute directly to the progression of gastric cancer. Further investigation of the molecular mechanisms of adhesive tumour-host interactions may lead to a better understanding of the natural history of gastric cancer.


British Journal of Cancer (1998) 78(2), 267–271
© 1998 Cancer Research Campaign

### Circulating intercellular adhesion molecule-1 and E-selectin levels in gastric cancer

**M Benekli, IH Güllü, G Tekuzman, MC Savaş, M Hayran, G Hasçelik and D Firat**

Institute of Oncology, Hacettepe University School of Medicine, Ankara, Turkey

**Summary** A diversity of adhesive interactions occur between the cancer cell and host extracellular matrix which potentiate neoplastic expansion and metastatic dissemination. In miscellaneous malignant diseases, tumour progression has been observed to be associated with alterations in adhesion molecule expression. Recently, circulating soluble intercellular adhesion molecules have been identified. In this study, serum levels of soluble intercellular adhesion molecule-1 (sICAM-1) and soluble E-selectin (sE-selectin) were determined in patients with gastric cancer. The study group consisted of 27 patients with previously untreated gastric adenocarcinoma. Four patients had stage II, two patients stage III and 21 patients stage IV disease according to the TNM classification. Nineteen patients had distant metastasis. The sera obtained from 18 healthy volunteers served as controls. Serum sICAM-1 and sE-selectin concentrations were determined by enzyme-linked immunosorbent assay (ELISA). In addition, we also studied other tumour-associated antigens, i.e. CEA and CA 19-9. Serum sICAM-1 levels were significantly increased in patients with gastric cancer ($P < 0.0001$). However, sE-selectin levels did not differ from the controls. sICAM-1 concentrations were also significantly higher in patients with distant metastasis and peritoneal spread ($P = 0.0045$ and $P = 0.0157$ respectively), whereas sE-Selectin levels were elevated only in patients with peritoneal metastasis ($P = 0.033$). Serum concentrations of sICAM-1 and sE-selectin correlated with CEA levels ($P = 0.0013$ and $P = 0.003$ respectively). Elevated levels of sE-selectin were associated with poorer prognosis ($P = 0.0099$), whereas sICAM-1 had no significant impact on survival. Our results suggest that increased sICAM-1 serum levels may reflect widespread disease and contribute directly to the progression of gastric cancer. Further investigation of the molecular mechanisms of adhesive tumour–host interactions may lead to a better understanding of the natural history of gastric cancer.

**Keywords:** circulating adhesion molecule; ICAM-1; E-selectin; gastric cancer

The outcome of patients with stomach cancer is extremely poor. Most cases are diagnosed at an advanced stage and the five-year survival rate is approximately 5–15% (Silverberg et al, 1992). The major determinant of survival following gastric cancer appears to be the development of metastases. Identification of biomolecules involved in the progression and dissemination of gastric cancer has gained considerable interest in the recent decade. Attachment factors, proteinases, natural proteinase inhibitors and motility factors are being investigated in patients with gastric cancer (Honda et al, 1996; Schwartz, 1996).

Adhesion molecules are distinct membrane surface receptors that participate in coordinating vital biological events such as morphogenesis, cell migration and intercellular communication (Springer, 1990; Carlos and Harlan, 1994; Frenette and Wagner, 1996*a*,*b*). There is substantial evidence to suggest that cell–cell and cell–matrix adhesive interactions play a crucial role in tumorigenesis, tumour progression and in particular metastasis (Albelda, 1993; Juliano, 1987; Pauli et al, 1990; Tuszynski et al, 1997). The expression, function and regulation of adhesion molecules are essential in these complex events and dysfunction or dysregulation of their equilibrium consequently results in disruption of normal cellular architecture and differentiation.

Intercellular adhesion molecule-1 (ICAM-1, CD54) is a member of the immunoglobulin supergene family of adhesion proteins which serves as the counter-receptor for a leucocyte integrin adhesion receptor, lymphocyte function-associated antigen-1 (LFA-1) (Marlin and Springer, 1987). The interaction of ICAM-1 and LFA-1 is important in the pathophysiology of the disease, including tumour progression and metastasis. ICAM-1 has been shown to be expressed on malignant cells in a number of haematological and non-haematological neoplasms.

Selectins are adhesion molecules that mediate the initial binding of leucocytes to microvascular endothelium by lectin-type interactions with carbohydrate ligands on corresponding target cells (Bevilacqua et al, 1991; Bevilacqua and Nelson, 1993). E-selectin (CD62E) is detected on the surfaces of endothelial cells upon activation by cytokines and hence designated by prefix E-, which stands for endothelium. E-selectin binds to target cell surfaces by oligosaccharide recognition, specifically to sialyl-Lewis x (sLe$^x$). sLe$^x$ is not responsible for all E-selectin-mediated adhesion processes, and the sLe$^a$ moiety (also known as CA 19–9), which has been shown to be expressed on adenocarcinomas of gastrointestinal tract, is also involved in E-selectin-mediated adhesion (Majuri et al, 1992).

Adhesion molecules can be detected in soluble forms in the circulation, and raised levels have been reported in several conditions, including different neoplastic conditions, higher concentrations being associated with liver metastases in gastrointestinal cancers (Harning et al, 1991; Tsujisaki et al, 1991; Banks et al, 1993; Gearing and Newman, 1993; Pui et al, 1993; Christiansen et al, 1996). However, effect on survival was determined only in patients with malignant melanoma, lymphomas and Hodgkin's disease (Harning et al, 1991; Pui et al, 1993; Christiansen et al, 1996).

*Received 1 October 1997*
*Revised 29 December 1997*
*Accepted 31 December 1997*

*Correspondence to:* M Benekli, 14 Sokak, 43/3, 06490, Bahçelievler, Ankara, Turkey

In this study, we analysed serum levels of sICAM-1 and sE-selectin in patients with newly diagnosed gastric carcinoma and determined their relation with metastasis and effect on survival.

### PATIENTS AND METHODS

### Patients and controls

Serum samples were collected from 27 patients with newly diagnosed gastric cancer between June 1995 and February 1996 at the Department of Medical Oncology, Hacettepe University Institute of Oncology, Ankara, Turkey. There were 18 males and nine females with a median age of 60 years (range 33–75). All patients had histologically proven gastric adenocarcinoma. Patient characteristics are shown in Table 1. Staging was carried out according to the revised American Joint Committee on Cancer (AJCC) International TNM system, which is based on post-gastrectomy pathological staging. Four patients had stage II, two patients stage III and 21 patients stage IV disease. Nineteen of the stage IV patients had distant organ-tissue metastases and the remaining two had locally advanced disease. Eleven patients had peritoneal dissemination, whereas seven patients could not be evaluated for peritoneal involvement. Distant organ and lymph node involvement were documented by chest radiography, abdominal ultrasonography and computerized tomography. Endoscopic sonography, bone scanning and bone marrow aspiration and biopsy were performed, if needed, in selected patients. Surviving patients were followed for about one year with a median of 4 months.

The sera obtained from 18 age- and sex-matched healthy hospital personnel, including 11 men and seven women, served as controls. Their median age was 51 years (range 24–70 years).

### Collection and storage of serum samples

Blood samples were collected following non-traumatic venepuncture before the surgery and/or chemotherapy and allowed to clot at room temperature for 2 h. The aliquots were separated following centrifugation for 5 min at 2500 r.p.m. and stored at –30°C until assayed.

### Assay of circulating sICAM-1 and sE-selectin

Serum levels of circulating adhesion molecules sICAM-1 and sE-selectin were measured with commercial sandwich ELISA assays (R&D Systems Europe, UK) according to the manufacturer's instructions. In brief, microtitre plates were coated with a monoclonal antibody against one epitope on sICAM-1 and sE-selectin. Standards and samples were transferred to the coated microtitre plates and incubated with streptavidin–horseradish peroxidase conjugate and a biotinylated antibody recognizing a second epitope on sICAM-1 and sE-selectin. A colour reaction was developed with tetramethylbenzidine and the absorbance was read at 450 nm with a correction wavelength of 620 nm.

### Measurement of carcinoembryonic antigen and carbohydrate antigen 19-9

We also simultaneously measured the levels of CEA and CA 19-9, which are established tumour markers for gastric cancer, using commercially available IMMULITE CEA and CA 19-9 kits (DPC, CA, USA). CEA is also an adhesion molecule similar in structure to the immunoglobulin superfamily. The recommended cut-off values for CEA and CA 19-9 were 5 ng ml$^{-1}$ and 37 U ml$^{-1}$ respectively.

**Table 1** Patient characteristics

| Characteristic | Number |
|---|---|
| Patients | 27 |
| Male/female | 18/9 |
| Median age in years (range) | 60 (33–75) |
| Stage | |
| I | 0 |
| II | 4 |
| III (A+B) | 2 |
| IV | 21 |
| Tumour location | |
| Antrum | 13 |
| Corpus | 9 |
| Cardia | 5 |
| Metastatic disease | 19 |
| Peritoneum | 11 |
| Liver | 11 |
| Bone | 4 |
| Bone marrow | 2 |
| Lung | 2 |
| Performance status (ECOG) | |
| 0 | 4 |
| 1 | 6 |
| 2 | 9 |
| 3 | 3 |
| 4 | 5 |

### Statistical analysis

Data are presented as median and interquartile range [median (IQR; 75th–25th percentile)]. *P*-values < 0.05 were assigned to be significant. Intergroup adhesion molecule concentration comparisons were performed by means of the Mann–Whitney *U*-test. For correlations between sICAM-1 and sE-selectin and other tumour-associated antigens, the Spearman's rank correlation test was utilized. Cut-off levels of sICAM-1 and sE-selectin values were determined using 'receiver operator characteristics' (ROC) analysis. Sensitivity, specificity and positive and negative predictive values for metastasis were calculated. Survival analysis was estimated by the Kaplan–Meier method and examined by the log-rank test. Data were analysed using 'Statistical Package for Social Sciences (SPSS) v 5.01 for Windows' computer program.

### RESULTS

### Concentrations of circulating sICAM-1 and sE-selectin in gastric cancer

Serum concentrations of sICAM-1 in gastric cancer patients were significantly elevated, whereas sE-selectin levels did not differ from those in normal controls. sICAM-1 and sE-selectin levels were 408.4 (202.2) ng ml$^{-1}$vs 279.85 (95.80) ($P < 0.0001$) and 66.0 (46.0) ng ml$^{-1}$ vs 59.95 (32.40) ng ml$^{-1}$ ($P = 0.4584$) respectively (Figure 1).

As shown in Table 2, serum levels of sICAM-1 were significantly increased in gastric cancer patients with distant organ

© Cancer Research Campaign 1998

**Figure 1** Serum levels of sICAM-1 and sE-selectin in gastric cancer patients and controls and/or tissue metastases [441.60 (374.4) ng ml$^{-1}$] compared with patients without metastases [304.4 (88.3) ng ml$^{-1}$] ($P$ = 0.0045). sE-selectin levels appeared to be elevated in metastatic gastric cancer patients [70.0 (38.0) ng ml$^{-1}$] compared with patients without distant metastases [55.0 (38.0) ng ml$^{-1}$]; however, the differences did not reach statistical significance ($P$ = 0.144) (Figure 2).

Adhesion molecule levels were also evaluated according to peritoneal involvement (Table 2). Higher levels of sICAM-1 were noted in patients with peritoneal spread [441.60 (174.6) ng ml$^{-1}$] than in patients without peritoneal implants [296.0 (108.8) ng ml$^{-1}$] ($P$ = 0.0157). In patients with peritoneal involvement, sE-selectin concentrations were also significantly raised compared with patients without peritoneal metastases [84.0 (34.0) ng ml$^{-1}$ vs 51.0 (34.0) ng ml$^{-1}$; $P$ = 0.033] (Figure 3).

**Table 2** Serum concentrations (ng ml$^{-1}$) of sICAM-1 and sE-selectin in patients with gastric cancer

| | sICAM-1 | P | sE-selectin | P |
|---|---|---|---|---|
| | Median (IQR) | | Median (IQR) | |
| Gastric cancer ($n$ = 27) | 408.4 (202.2) | 0.0001 | 66.0 (46.0) | 0.4584 |
| Controls ($n$ = 18) | 279.85 (95.80) | | 59.95 (32.40) | |
| Distant metastasis | | | | |
| Present ($n$ = 19) | 441.60 (374.4) | 0.0045 | 70.0 (38.0) | 0.144 |
| Absent ($n$ = 8) | 304.4 (88.3) | | 55.0 (38.0) | |
| Peritoneal metastasis | | | | |
| Present ($n$ = 11) | 441.60 (174.6) | 0.0157 | 84.0 (34.0) | 0.033 |
| Absent ($n$ = 9) | 296.0 (108.8) | | 51.0 (34.0) | |

### Correlation of sICAM-1 and sE-selectin with tumour-associated antigens

Serum levels of sICAM-1 and sE-selectin did not correlate with each other, nor with serum CA 19-9 levels (data not shown). A significant correlation was, however, found between CEA serum levels and the levels of both adhesion molecules sICAM-1 and sE-selectin (moderate positive correlation, $r$ = 0.50 and $P$ = 0.0013 vs $r$ = 0.57 and $P$ = 0.003 respectively).

### Prognosis and the serum adhesion molecule levels

ROC curve analysis revealed the presence of significant cut-off values for sICAM-1 ($z$ = 4.42, one-tailed $P$ < 0.001) and sE-selectin ($z$ = 1.88, one-tailed $P$ = 0.031) with reasonable predictive values for metastasis. Cut-off levels for sICAM-1 and sE-selectin were determined as 380 ng ml$^{-1}$ and 70 ng ml$^{-1}$ respectively. Sensitivity, specificity and positive and negative predictive values were calculated according to the cut-off values (Table 3). Gastric cancer patients with serum sE-selectin levels over 70 ng ml$^{-1}$ proved to have a significantly shorter overall survival than those with lower serum concentrations ($P$ = 0.0099) (Figure 4). Serum sICAM-1 levels did not significantly affect survival ($P$ = 0.1390). Early death of the majority of the patients during follow-up might be responsible for this unexpected result. Finally, higher CEA levels, but not CA 19-9 levels, indicated worse prognosis ($P$ = 0.0408 vs $P$ = 0.2080).

### DISCUSSION

Neoplastic transformation and the evolution to metastatic disease are characterized by a dramatic aberration in cellular cohesive

© Cancer Research Campaign 1998 

**Figure 2** Serum sICAM-1 and sE-selectin levels in metastatic and non-metastatic gastric cancer patients. Error bars represent the dispersion of sICAM-1 and sE-selectin values. Edges of each box correspond to 25th and 75th percentiles and the thicker middle bars indicate the medians

**Figure 3** Serum sICAM-1 and sE-selectin levels in gastric cancer patients with or without peritoneal metastasis. Error bars represent the dispersion of sICAM-1 and sE-selectin values. Edges of each box correspond to 25th and 75th percentiles and the thicker middle bars indicate the medians

**Figure 4** Patient survival according to sICAM-1 (**A**) and sE-selectin (**B**) levels interactions (Albelda, 1993; Juliano, 1987; Pauli et al, 1990; Tuszynski et al, 1997). Adhesion proteins are involved in many of the intermediate steps of metastatic cascade and are likely to show pronounced changes in expression during malignant progression. Tumour cells invade the surrounding connective tissue and are liberated away from their primary localization after the disruption of connections between neighbouring cells. Circulating tumour clusters adhere selectively to the microvascular endothelium of the selected secondary target organ site. This process is suggested to be mediated by organ-specific adhesion molecules, which are expressed on the endothelial cells of the preferred site and which serve as 'homing receptors' (Pauli et al, 1990). These adhesion molecules have also been shown to facilitate tumour cell motility and, therefore, enhance the invasion of tumour cells into the tissue parenchyma at the metastatic site.

Malignant cells possess many membrane surface antigens that regulate fundamental cellular functions, including the MHC antigens, differentiation antigens, tumour-specific receptors and tumour-associated antigens (Black, 1980). These antigens are shed into the circulation and usually reflect the extent of the disease. Molecules mediating leucocyte–endothelium adhesion may serve as tumour-associated antigens in a variety of solid tumours (Black, 1980). ICAM-1, together with MHC antigens, plays an important role in the human immune response, involving T-cell activation and other lymphocyte effector functions. ICAM-1 and MHC antigens are frequently coexpressed on the gastric carcinoma cells which may suggest a possible contribution of the immune system to the dissemination of the tumour (Koyama et al, 1992; Ishii et al, 1994). Moreover, adhesion molecule shedding from the surface of the cancer cell may represent an important mechanism for tumour cells to escape immunosurveillance.

In this report, we have demonstrated that concentrations of sICAM-1 are increased in gastric cancer, especially in patients with distant organ and peritoneal metastases. sE-selectin levels did not differ from the controls in patients with gastric cancer and in metastatic patients, but were elevated significantly in patients with

**Table 3** sICAM-1, sE-selectin, CEA and CA 19-9 sensitivity, specificity, positive and negative predictive values

| | Cut-off (ng ml$^{-1}$) | Sensitivity (%) | Specificity (%) | PPV (%) | NPV (%) |
|---|---|---|---|---|---|
| sICAM-1 | 380 | 73.7 | 87.5 | 93.3 | 53.8 |
| sE-selectin | 70 | 47.4 | 87.5 | 90.0 | 41.2 |
| CEA | 5.5 | 51.7 | 94.4 | 93.3 | 56.6 |
| CA 19-9 (U ml$^{-1}$) | 33 | 55.5 | 88.9 | 88.2 | 57.1 |

PPV, positive predictive value; NPV, negative predictive value.

 © Cancer Research Campaign 1998

peritoneal metastases. For sICAM-1, these findings confirm data published previously in the literature. However, in contrast, only higher (> 70 ng $ml^{-1}$) sE-selectin levels indicated worse prognosis in survival analysis, although sICAM-1 levels had no significant impact on survival. Serum levels of sICAM-1 and sE-selectin were correlated with serum CEA levels, but not with CA 19-9 levels. The significant correlations found between sICAM-1 or sE-selectin and CEA probably reflect the similar nature of these molecules, which function as adhesion molecules.

Little is known about the expression and functions of adhesion molecules in gastric cancer. ICAM-1 was shown to be expressed on the surface of primary and metastatic gastric carcinoma cells (Koyama et al, 1992; Ishii et al, 1994; Mayer et al, 1995; Nasu et al, 1997), but no correlation with survival was found (Mayer et al, 1995). Mizoi et al (1995) described positive staining of stromal macrophages with ICAM-1 and of lymphocytes with LFA-1 along the invasive margin of gastric cancer, and suggested a possible role of ICAM-1/LFA-1 interaction in host immune/inflammatory reaction.

Contradictory results have also been reported in the literature. Ura et al (1996), in contrast to our study, demonstrated that the expression of ICAM-1 was inversely correlated with liver metastasis. Yasoshima et al (1996) also showed a reduced expression of ICAM-1 and of LFA-1 and increased surface expression of $\beta 1$ integrins in an experimental animal model of high-metastatic gastric cancer cell line.

Data on circulating adhesion molecules in gastric cancer are insufficient. Our results confirm and augment data reported by Tsujisaki et al (1991), who detected increased levels of sICAM-1 in the sera of gastric cancer patients, with particularly higher levels in patients with liver metastases. They also demonstrated that both expression and shedding of ICAM-1 antigen increased when cultured gastric carcinoma cells were treated with interferon-$\gamma$. Banks et al (1993) examined the serum concentrations of adhesion molecules sE-selectin, sICAM-1 and cVCAM-1 in randomly selected cancer patients. The levels of all three adhesion receptors were found to be elevated in patients with gastrointestinal cancer, but the exact localization of the tumours were not specified. Moreover, the significance of adhesion molecule levels in metastatic disease was not determined, but it was stated that 85% of all cases with epithelial tumours were metastatic. The prognostic significance of adhesion molecules were not determined in either study.

In conclusion, the results of this study provide strong circumstantial evidence that alterations in the expression and function of adhesion molecules may play a critical role in the aggressive behaviour of gastric cancer. We have shown that the serum concentrations of sICAM-1 are significantly elevated in gastric cancer. The level of sICAM-1 reflected metastatic dissemination and correlated with a well-known tumour-associated antigen, CEA. The functional role and pathophysiological consequences of elevated levels of sICAM-1 need to be further investigated. The identification of the peculiar mechanisms of the adhesion molecule aberrations may lead to a better understanding of the natural history of gastric cancer and also of other solid epithelial malignancies.

### REFERENCES

Albelda SM (1993) Biology of disease. Role of integrins and other adhesion molecules in tumor progression and metastasis. *Lab Invest* **68**: 4–17

Banks RE, Gearing AJH, Hemingway IK, Norfolk DR, Perren TJ and Selby PJ (1993) Circulating intercellular adhesion molecule-1 (ICAM-1), E-selectin, and vascular cell adhesion molecule-1 (VCAM-1) in human malignancies. *Br J Cancer* **68**: 122–124

Bevilacqua M, Butcher E, Furie B, Furie B, Gallatin M, Gimbrone M, Harlan J, Kishimoto K, Lasky L, McEver R, Paulson J, Rosen S, Seed B, Siegelman M, Springer T, Stoolman L, Tedder T, Varki A, Wagner D, Weissman I and Zimmerman G (1991) Selectins: a family of adhesion receptors. *Cell* **67**: 233

Bevilacqua MP and Nelson RM (1993) Selectins. *J Clin Invest* **91**: 379–387

Black PH (1980) Shedding from the cell surface of normal and cancer cells. *Adv Cancer Res* **32**: 75–199

Carlos TM and Harlan JM (1994) Leukocyte-endothelial adhesion molecules. *Blood* **84**: 2068–2101

Christiansen I, Gidlöf C and Kalkner K-M (1996) Elevated serum levels of soluble ICAM-1 in non-Hodgkin's lymphomas correlate with tumour burden, disease activity and other prognostic markers. *Br J Haematol* **92**: 639–636

Frenette PS and Wagner DD (1996*a*) Adhesion molecules, Part I. *New Engl J Med* **334**: 1526–1529

Frenette PS and Wagner DD (1996*b*) Adhesion molecules, Part II. *New Engl J Med* **335**: 43–45

Gearing AJH and Newman W (1993) Circulating adhesion molecules in disease. *Immunol Today* **14**: 506–512

Harning R, Mainolfi E, Bystryn J-C, Henn M, Merluzzi VJ and Rothlein R (1991) Serum levels of intercellular adhesion molecule-1 in human malignant melanoma. *Cancer Res* **51**: 5003–5005

Honda M, Mori M, Ueo H, Sugimachi K and Akiyoshi T (1996) Matrix metalloproteinase-7 expression in gastric carcinoma. *Gut* **39**: 444–448

Ishii H, Gouchi A and Orita K (1994) The enhancement of cell surface ICAM-1 and HLA class 1 antigens in human gastric cancer cell lines by IFN-gamma. *Acta Med Okayama* **48**: 73–79

Juliano RL (1987) Membrane receptors for extracellular matrix macromolecules: relationship to cell adhesion and tumor metastasis. *Biochim Biophys Acta* **907**: 261–278

Koyama S, Ebihara T and Fukao K (1992) Expression of intercellular adhesion molecule-1 (ICAM-1) during the development of invasion and/or metastasis of gastric carcinoma. *J Cancer Res Clin Oncol* **118**: 609–614

Majuri M-L, Mattila P and Renkonen R (1992) Recombinant E-selectin-protein mediates tumor cell adhesion via sialyl-Lea and sialyl-Lex. *Biochem Biophys Res Commun* **182**: 1376–1382

Marlin SD and Springer TA (1987) Purified intercellular adhesion molecule-1 (ICAM-1) is a ligand for lymphocyte function-associated antigen 1 (LFA-1). *Cell* **51**: 813–819

Mayer B, Lorenz C, Babic R, Jauch KW, Schildberg FW, Funke I and Johnson JP (1995) Expression of leukocyte cell adhesion molecules on gastric carcinomas: possible involvement of LFA-3 expression in the development of distant metastases. *Int J Cancer* **64**: 415–423

Mizoi T, Ohtani H, Suzuki Y, Shiiba K, Matsuno S and Nagura H (1995) Intercellular adhesion molecule-1 expression by macrophages in human gastrointestinal carcinoma: possible roles as host immune/inflammatory reaction. *Pathol Int* **45**: 565–572

Nasu R, Mizuno M, Kiso T, Shimo K, Uesu T, Nasu J, Tomoda J, Okada H and Tsuji T (1997) Immunohistochemical analysis of intercellular adhesion molecule-1 expression in human gastric adenoma and adenocarcinoma. *Virchows Arch* **430**: 279–283

Pauli BU, Augustin-Voss HG, El-Sabban ME, Johnson RC and Hammer DA (1990) Organ preference of metastasis: the role of endothelial cell adhesion molecules. *Cancer Metastasis Rev* **9**: 175–189

Pui C-H, Luo X, Evans W, Martin S, Rugg A, Wilimas J, Crist WM and Hudson M (1993) Serum intercellular adhesion molecule-1 in childhood malignancy. *Blood* **82**: 895–898

Schwartz GK (1996) Invasion and metastases in gastric cancer: in vitro and in vivo models for clinical correlations. *Semin Oncol* **23**: 316–324

Silverberg E, Boring CC and Squires TS (1992) Cancer statistics. *CA Cancer J Clin* **42**: 1–38

Springer TA (1990) Adhesion receptors of the immune system. *Nature* **346**: 425–434

Tsujisaki M, Imai K, Hirata H, Hanzawa Y, Masuya J, Nakano T, Sugiyama T, Matsui M, Hinoda Y and Yachi A (1991) Detection of circulating intercellular adhesion molecule-1 antigen in malignant diseases. *Clin Exp Immunol* **85**: 3–8

Tuszynski GP, Wang TN and Berger D (1997) Adhesive proteins and the hematogenous spread of cancer. *Acta Haematol* **97**: 29–39

Ura H, Denno R and Hirata K (1996) Correlation between nm23 protein and several cell adhesion molecules in human gastric carcinoma. *Jpn J Cancer Res* **87**: 512–517

Yasoshima T, Denno R, Kawaguchi S, Sato N, Okada Y, Ura H, Kikuchi K and Hirata K (1996) Establishment and characterisation of human gastric carcinoma lines with high metastatic potential in the liver: changes in integrin expression associated with the ability to metastasize in the liver of nude mice. *Jpn J Cancer Res* **87**: 153–160

© Cancer Research Campaign 1998